# Analysis of the nucleotide sequence of the guinea pig cytomegalovirus (GPCMV) genome

**DOI:** 10.1186/1743-422X-5-139

**Published:** 2008-11-12

**Authors:** Mark R Schleiss, Alistair McGregor, K Yeon Choi, Shailesh V Date, Xiaohong Cui, Michael A McVoy

**Affiliations:** 1 Center for Infectious Diseases and Microbiology Translational Research, University of Minnesota, 2001 6th Street SE, Minneapolis, MN 55455, USA; 2 Genentech, Inc., 1 DNA Way, South San Francisco, CA 94080, USA; 3 Division of Infectious Diseases, Department of Pediatrics, Virginia Commonwealth University School of Medicine, P.O. Box 163, MCV Station, Richmond, VA 23298, USA

## Abstract

In this report we describe the genomic sequence of guinea pig cytomegalovirus (GPCMV) assembled from a tissue culture-derived bacterial artificial chromosome clone, plasmid clones of viral restriction fragments, and direct PCR sequencing of viral DNA. The GPCMV genome is 232,678 bp, excluding the terminal repeats, and has a GC content of 55%. A total of 105 open reading frames (ORFs) of > 100 amino acids with sequence and/or positional homology to other CMV ORFs were annotated. Positional and sequence homologs of human cytomegalovirus open reading frames *UL23 *through *UL122 *were identified. Homology with other cytomegaloviruses was most prominent in the central ~60% of the genome, with divergence of sequence and lack of conserved homologs at the respective genomic termini. Of interest, the GPCMV genome was found in many cases to bear stronger phylogenetic similarity to primate CMVs than to rodent CMVs. The sequence of GPCMV should facilitate vaccine and pathogenesis studies in this model of congenital CMV infection.

## Findings

Guinea pig cytomegalovirus (GPCMV) serves as a useful model of congenital infection, due to the ability of the virus to cross the placenta and infect the fetus *in utero *[1-3]. This model is well-suited to vaccine studies for prevention of congenital cytomegalovirus (CMV) infection, a major public health problem and a high-priority area for new vaccine development [4]. However, an impediment to studies in this model has been the lack of detailed DNA sequence data. Although a number of reports have identified specific gene products or clusters of genes [5-11], to date a full genomic sequence has not been available.

We recently reported the construction and preliminary sequence map of a GPCMV bacterial artificial chromosome (BAC) clone maintained in *E. coli *[12,13], and this clone was used as an initial template for sequence analysis of the full GPCMV genome. BAC DNA was purified using Clontech's NucleoBond^® ^Plasmid Kits as described previously [14] and both strands were sequenced using an ABI PRISM^® ^377 DNA Sequencer, with primers synthesized, as needed, to 'primer-walk' the nucleotide sequence. In parallel, *Hin*d III- and *EcoR *I-digested fragments were gel-purified and cloned into pUC and pBR322-based vectors as previously described [15]. Plasmid sequences were determined from overlapping *Hin*d III and *EcoR *I fragments using the map coordinates originally described by Gao and Isom [16]. These sequences were compared to the BAC sequence to facilitate assembly of a full-length contiguous sequence. Since the cloning of the BAC in *E. coli *involved insertion of BAC origin sequences into the *Hin*d III "N" region of the viral genome, sequence obtained from this specific restriction fragment cloned in pBR322 was utilized for assembly of the final contiguous sequence; analysis of this sequence confirmed that there were no adventitious deletions in the *Hin*d III "N" region generated during the original BAC cloning process. Since a deletion in the *Hin*d III "D" region occurred during cloning of the GPCMV BAC in *E. coli *[17], DNA sequence from a plasmid containing the full-length *Hin*d III "D" fragment was similarly obtained, and used for assembly of the final contiguous sequence. The GPCMV genomic sequence has been deposited with GenBank (Accession Number FJ355434).

Sequence analysis of GPCMV revealed a genome length of 232,678 bp with a GC content of 55%. This value is in agreement with the value of 54.1% determined previously by CsCl buoyant density centrifugation [18]. A total of 326 open reading frames (ORFs) were identified that were capable of encoding proteins of ≥ 100 amino acids (aa). For ORFs predicted by the sequence analysis that had substantial overlap with other adjacent or complementary GPCMV ORFs that appeared to encode gene products that were highly conserved in other cytomegaloviruses, only those sequences with < 60% overlap with these highly conserved ORFs were further analyzed. ORFs homologous to those encoded by other CMVs with an e-value of < 0.1 and ≥ 100 aa were identified, based on comparisons analyzed using NCBI Blast (blastall version program 2.2.16). Of the ORFs so identified, 104 had sequence and/or positional homology to one or more ORFs encoded by human (HCMV), murine (MCMV), rat (RCMV), rhesus (RhCMV), chimpanzee (CCMV), or tupaia herpesvirus (THV) cytomegaloviruses (Table 1). Of note, homologs of HCMV ORFs *UL23 *through *UL122 *were identified [19]. For ease of nomenclature, we have designated these ORFs using upper case font (*GP23 *through *GP122*). ORFs with homologs in other CMVs that do not correspond to HCMV *UL23 *through *UL122 *have been designated with a lower case "gp" prefix. Homologs of HCMV *UL41a* (69 aa; *gp38.2*), *UL51* (99 aa; *GP51*), and *UL91* (87 aa; *GP91*) were annotated in these initial analyses, based primarily on positional, and not sequence, homology to the respective HCMV ORFs.  Three ORFs, homologs of MHC class I genes known to be encoded by multiple other CMVs (gp 147–149, Table 1) were also identified.  One ORF, gp1 (homolog of CC chemokines), did not have a positional or sequence homolog when compared to other CMVs, but was included in the annotation because of its previous molecular characterization  [9].  Including ORFs with mapped exons, the total number of ORFs annotated in this preliminary analysis was 105 [Table 1].      

**Table 1:** GPCMV Open Reading Frames (ORFs)

**ORF**	**Strand**	**Position**		**Size (aa)**	**Protein Characteristics and Cytomegalovirus Homologs**
				
		**From**	**To**		
gp1	C	12701	13006	101	GPCMV MIP 1-alpha; homology to multiple CC chemokines

gp2		15098	15949	283	Homology to MCMV M69^a^

gp3	C	17461	19827	788	Homology to THV T5^b^; US22 superfamily

gp4	C	21093	21416	107	Homology to RCMV r136^d^

gp5	C	26985	28097	370	Homology to MCMV m32^a^

gp6		30089	30454	121	Homology to MCMV glycoprotein family m02^a^

gp7	C	32003	32308	101	Homology to RhCMV rh42^c^

**GP23**	C	33561	34763	400	UL23 homolog; US22 gene superfamily

**GP24**	C	35000	36217	405	UL24 homolog; US22 superfamily

gp24.1		36802	37224	140	Homology to MCMV M34 protein^a^

**GP25**		37187	38455	422	UL25 homolog; tegument protein

**GP26**	C	38621	39058	145	UL26 homolog

**GP27**	C	39508	41472	654	UL27 homolog

**GP28**	C	41572	42639	355	UL28 homolog; US22 superfamily

**GP28.1**	C	43344	44546	400	UL28 homolog; US22 superfamily

**GP28.2**	C	44912	46099	395	UL28 homolog; US22 superfamily

**GP29**	C	46211	46882	223	UL29 homolog; US22 superfamily

gp29.1	C	47579	48034	151	Homology to RCMV R36 protein^d^; potential homolog of viral cell death suppressor

**GP30**	C	49363	51060	565	UL30 homolog

**GP31**		51354	52832	492	UL31 homolog

**GP32**	C	53073	54626	518	UL32 homolog

**GP33**		54846	56129	427	UL33 homolog; 7-TMR GPCR superfamily

**GP34**		56482	58065	527	UL34 homolog

**GP35**		58269	59927	552	UL35 homolog

**GP37**	C	60047	60964	305	UL37 homolog

**GP38**	C	61321	62385	354	UL38 homolog

gp38.1	C	62960	63817	436	Positional homolog of HCMV UL40

gp38.2	C	63876	65186	69	Positional homolog of HCMV UL41a

gp38.3	C	65881	66735	284	Positional homolog of HCMV UL42

gp38.4	C	67254	67619	121	Homology to RCMV r42^d^

**GP43**	C	68208	69221	337	UL43 homolog

**GP44**	C	69209	70432	407	UL44 homolog

**GP45**	C	71144	73933	929	UL45 homolog

**GP46**	C	74036	74833	265	UL46 homolog

**GP47**		75441	77846	801	UL47 homolog

**GP48**		78051	84332	2093	UL48 homolog

**GP49**	C	84746	86386	546	UL49 homolog

**GP50**	C	86362	87426	354	UL50 homolog

**GP51**	C	87551	87850	99	UL51 homolog; terminase subunit

**GP52**		88170	89750	526	UL52 homolog

**GP53**		89743	90729	328	UL53 homolog

**GP54**	C	90821	94174	1117	UL54 homolog; DNA polymerase

**GP55**	C	94216	96921	901	UL55 homolog; glycoprotein B

**GP56**	C	96818	99085	755	UL56 homolog; terminase subunit

**GP57**	C	99236	102919	1227	UL57 homolog

gp57.1	C	104872	105258	128	Homology to RCMV r23.1^d^

gp57.2		107338	107712	124	Homology to RCMV R53^d^

**GP69**	C	108547	111678	1043	UL69 homolog

**GP70**	C	112387	115590	1067	UL70 homolog; helicase-primase

**GP71**		115589	116365	258	UL71 homolog

**GP72**	C	116528	117601	357	UL72 homolog; dUTPase

**GP73**		117683	118084	133	UL73 homolog; glycoprotein N

**GP74**	C	118031	119143	370	UL74 homolog; glycoprotein O

**GP75**	C	119595	121766	723	UL75 homolog; glycoprotein H

**GP76**		121931	122770	279	UL76 homolog

**GP77**		122484	124343	619	UL77 homolog

**GP78**		124725	125969	414	UL78 homolog; 7-TMR GPCR superfamily

**GP79**	C	126164	127111	315	UL79 homolog

**GP80**		126972	129281	769	UL80 homolog; CMV protease

**GP82**	C	129576	131141	521	UL82 homolog; pp71

**GP83**	C	131361	133058	565	UL83 homolog; pp65

**GP84**	C	133286	134737	483	UL84 homolog

gp84.1		134994	135476	160	Homolog of RhCMV rh116^e^

**GP85**	C	135035	135946	303	UL85 homolog

**GP86**	C	136227	140276	1349	UL86 homolog

**GP87**		140657	143578	973	UL87 homolog

**GP88**		143481	144752	423	UL88 homolog

**GP89ex2**	C	144798	145928	376	UL89 homolog; terminase subunit, exon 2

**GP91**		146356	146619	87	UL91 homolog

**GP92**		146616	147245	209	UL92 homolog

**GP93**		147456	148985	509	UL93 homolog

**GP94**		149118	149873	251	UL94 homolog

**GP89ex1**	C	150285	151166	291	UL89 homolog; terminase subunit, exon 1

**GP95**		151284	152489	401	UL95 homolog

**GP96**		152722	153084	120	UL96 homolog

**GP97**		153164	154981	605	UL97 homolog; protein kinase

**GP98**		155001	156788	595	UL98 homolog; alkaline nuclease

**GP99**		156701	157222	173	UL99 homolog; pp28

gp99.1		157406	158020	204	Homology to RCMV r4^d^

**GP100**	C	157529	158578	349	UL100 homolog; glycoprotein M

**GP102**		158908	161193	761	UL102 homolog

**GP103**	C	161307	162104	265	UL103 homolog

**GP104**	C	162067	164160	697	UL104 homolog; portal

**GP105**		164000	166783	927	UL105 homolog; helicase-primase

gp105.1		176502	176894	130	Homology to RhCMV rh55^c^

**GP112ex1**		177066	177839	258	UL112 homolog; replication accessory protein, exon 1

**GP112ex2**		178403	179257	284	UL112/UL113 homolog; replication accessory protein, exon 2

**GP114**	C	179168	180259	363	UL114 homolog; uracil glycosylase

**GP115**	C	180325	181101	258	UL115 homolog; glycoprotein L

**GP116**	C	181146	181994	282	Homology to THV t116^b^; possible functional homolog of UL119; Fc receptor/immunoglobulin binding domains

**GP117**	C	182202	182777	191	UL117 homolog

**GP119.1**	C	185103	185591	162	UL119 homolog; homology to MCMV M119.1^a^

**GP121**	C	186635	187681	348	UL121 homolog; homology to THV t121.4^b^

**GP122**	C	188292	189260	322	UL122 homolog; HCMV IE2; immediate early transactivator

gp123		195838	196893	351	MCMV IE2 homolog^a^; US22 superfamily

gp138	C	201275	202750	491	Homology to RCMV r138^d^

gp139	C	204624	206717	697	Homology to THV T5^b^; US22 superfamily

gp140		206446	206853	135	Homology to CCMV UL132^g^

gp141	C	206977	208584	535	Homology to HCMV US23^h^; US22 superfamily

gp142	C	208852	210546	564	Homology to HCMV US24^h^; US22 superfamily

gp143	C	210799	212532	577	Homology to THV T5^b^; US22 superfamily

gp144	C	213034	215328	764	Homology to US26^h^; US22 gene superfamily

gp145	C	215601	217499	632	Homology to HCMV IRS1/TRS1^h^; US22 superfamily

gp146	C	218106	219839	577	Homology to HCMV IRS1/TRS1^h^; US22 superfamily

gp147	C	223464	225026	520	MHC class I homolog

gp148	C	225938	227389	483	MHC class I homolog

gp149	C	228845	230728	627	MHC class I homolog

^a ^Genbank NC_004065.1

^b ^Genbank NC_004065.1

^c ^Genbank NC_006150.1

^d ^Genbank AF232689.2

^e ^Genbank YP_068209.1

^f ^Genbank AY486477.1

^g ^Genbank NC_003521.1

^h ^Genbank NC_001347

**Figure 1:**
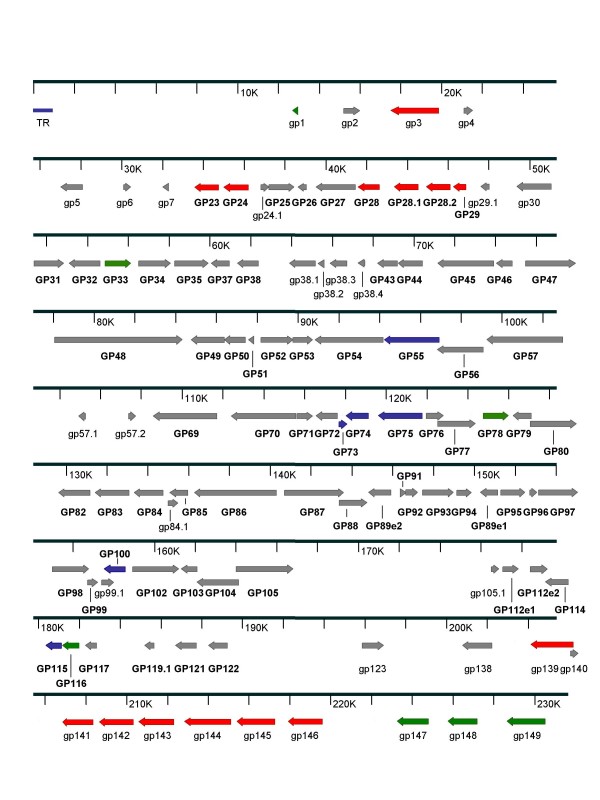
**Protein Coding Map of GPCMV Genome**. Schematic representation of the GPCMV genome demonstrating ORFs described in the text. GPCMV ORFs with positional and/or sequence homology to HCMV ORFs are indicated in bold with upper case prefixes (*GP23 *through *GP122*). ORFs that lack sequence or positional homologs in HCMV but share homology with ORFs in other CMVs are indicated with lower case prefixes (see Table 1). Only the 5' terminal repeat (TR) is shown; however, in about 50% of genomes the TR is duplicated at the 3' end [18]. Color-coding indicates ORFs of interest for vaccine and pathogenesis studies: blue, envelope glycoprotein homologs; green, putative immune evasion/immune modulation gene homologs; red, *US22 *superfamily homologs.

A map of the GPCMV genome illustrating the relative positions of these ORFs is shown in Fig. 1. ORFs that represent homologs of the individual exons of spliced HCMV genes, in particular *UL89 *(terminase) and *UL112/UL113 *(replication accessory protein) are annotated separately. The splice junction for the GP89 mRNA was predicted based on comparisons to other CMVs. For the UL112/113 region, further studies will be required to map the precise splicing patterns of the putative transcripts encoded by this region of the GPCMV genome. Similarly, the ORF encoding the sequence homolog of the HCMV IE transactivator, *UL122*, has been annotated without regard to the splicing events previously shown to take place in this region of the genome [20]; further analyses of cDNA from this and other GPCMV genome regions of IE transcription, including those encoded in the *Hin*d III 'D' region of the genome, will likely result in annotation of multiple heretofore unidentified ORFs. A comprehensive table of all ORFs > 25 aa and their homology to other CMV genomes is provided in additional files 1 and 2. As RNA analyses are completed, the total number of annotated GPCMV ORFs will expand in number.

The schematic representation of GPCMV ORFs demonstrated in Fig. 1 highlights several gene families of particular interest. Of particular interest and importance to vaccine studies in the guinea pig model are conserved homologs of the ORFs encoding major envelope glycoproteins gB, gH/gL/gO/, and gM/gN. These glycoproteins are important determinants of humoral immune responses in the setting of CMV infection, and serve as potential subunit vaccine candidates. Of these, the gB homolog has been demonstrated to confer protection against congenital GPCMV infection in subunit vaccine studies [21-23]. Homologs of putative HCMV immune modulation genes, including G-protein coupled receptors and major histocompatibility class I homologs, were also identified [24]. Also of interest was the presence of multiple *US22 *gene family homologs, heavily clustered near the rightward terminus of the GPCMV genome. These ORFs predict protein products that are analogous to the MCMV dsRNA-binding proteins, M142 and M143, that have been shown to inhibit dsRNA-activated antiviral pathways [25,26]. Members of this family have also been implicated in macrophage tropism in MCMV [27]. Our sequence analysis also confirmed the findings of Liu and Biegalke [8] that the GPCMV genome does not encode a positional homolog of the antiapoptotic HCMV *UL36 *gene [28]. However, an ORF with homology to *R36*, which encodes the presumed RCMV cell death suppressor, was identified (*gp29.1*, Table 1). Further studies will be required to determine whether this putative gene supplies a *UL36*-like function.

It was also of interest to note the presence of ORFs that have apparent homology to the MCMV *M129-133 *region. This region has positional homologs in human and primate CMVs [29-31], but is absent in THV [32]. Recently, it was determined that passage of GPCMV in cultured fibroblasts promotes the deletion of a ~1.6-kb locus containing potential positional homologs of this gene cluster. The presence of this 1.6 kb locus was found by Inoue and colleagues to be associated with an enhanced pathogenesis of GPCMV *in vivo *[33]. We independently confirmed the presence of this locus and its sequence in our salivary gland-derived viral stocks, and have included this sequence in our GenBank annotation (Accession Number FJ355434). Further studies will be required to fully annotate the transcripts encoded by this region of the GPCMV genome. Interestingly, the original GPCMV BAC clone that we sequenced was derived using GPCMV viral DNA obtained after long-term tissue culture passage of ATCC 2122 viral stock, and not surprisingly this BAC was found to lack the 1.6 kb virulence locus [12]. Subsequently, PCR and preliminary sequencing of a more recently obtained GPCMV BAC clone with an excisable origin of replication [17] revealed that the 1.6-kb sequence was retained in this clone. The apparent modifications of this locus that occur following viral passage on fibroblast cells are reminiscent of the mutations and deletions that occurred during fibroblast-passage of HCMV [34] and rhesus CMV [35]. The congruence of these events suggests that the selective pressures that promote mutational inactivation of genes in this region may be similar across viral species. Additional analyses, including sequencing of a full-length GPCMV genome derived from replicating virus *in vivo*, will be required to determine what other deletions or mutations are present in genomes from tissue culture-passaged viruses. Since additional ORFs are likely to be identified by these analyses, we have annotated the first ORF identified in the BAC sequence to the right of this 1.6 kb region as *gp138 *(Fig. 1), to allow for ease of nomenclature as ORFs in this virulence locus are better characterized. Application of other genome sequence analysis methods, including identification of small or overlapping genes and further assessment of mRNA splicing or unconventional translation signals, will likely result in identification of other putative ORFs in future studies [36].

**Figure 2:**
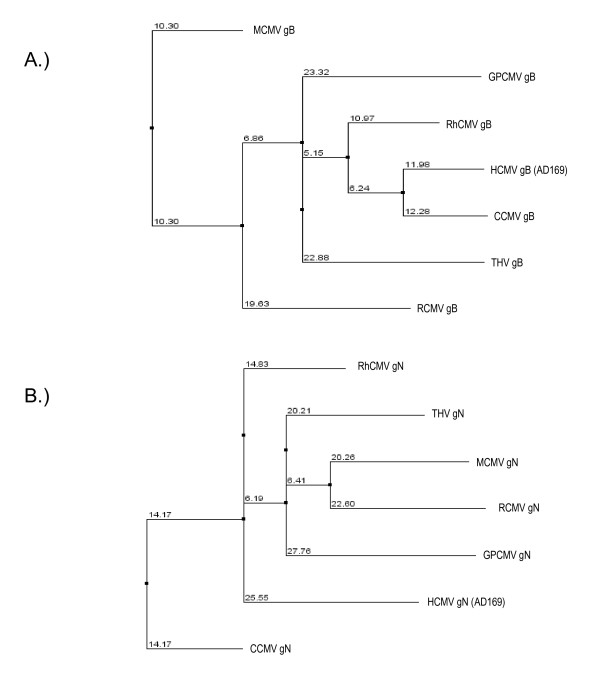
**Comparison of GPCMV Glycoproteins with CMV Homologs**. Sequences of GPCMV glycoproteins were aligned with glycoproteins from six other CMV genomes (HCMV, MCMV, RCMV, RhCMV, THV, and CCMV) using both ClustalW [37] and Muscle [38] using default parameters. Phylogenetic trees (neighbor joining) were generated from these alignments using Jalview. Numbers at each node indicate mismatch percentages. Interestingly, GPCMV sequences closely match THV sequences (see also, supplementary information), and generally appear closer to primate CMV glycoproteins in pair-wise comparisons than to rodent CMV glycoproteins, as previously observed for gB [39]. Clustal comparisons for conserved glycoproteins gB (GP55; Panel A) and gN (GP73; Panel B) are indicated.

Comparisons of GPCMV ORFs with sequences from other CMV genomes yielded interesting results. ORF translations were compared with all proteins from the 6 sequenced CMV genomes (HCMV, MCMV, RCMV, RhCMV, THV, and CCMV), and hits with e-values less than 1e^-5 ^were aligned individually for each protein, using both ClustalW (version 1.82; [37]) and Muscle (version 3.6; [38]). The alignments were then used to generate trees based on neighbor-joining using JalView. Clustal trees for glycoproteins B (*GP55*) and N (*GP73*) are shown in Fig. 2, with distance scores indicated. Overall, comparison of the various glycoproteins (gB, gM, gH, and gO) yielded similar phylogenies, with GPCMV glycoproteins generally appearing closer to primate CMVs than rodent CMVs [39], except for the gN homolog, which appears closer to rodents. ClustalW and Muscle comparisons of GPCMV ORFs with homologous ORFs from the other sequenced CMVs are provided in additional file 3.

In summary, the complete DNA sequence of GPCMV was determined, using a combination of sequencing of BAC DNA, viral DNA, and cloned *Hin*d III and *Eco*RI fragments. These analyses identified both conserved ORFs found in all mammalian CMVs, as well as the presence of novel genes apparently unique to the GPCMV. These similarities underscore the usefulness of the guinea pig model, with positive translational implications for development and testing of CMV intervention strategies in humans. Further characterization of the GPCMV genome should facilitate ongoing vaccine and pathogenesis studies in this uniquely useful small animal model of congenital CMV infection.

## Competing interests

The authors declare that they have no competing interest. SVD is an employee of Genentech Corporation.

## Authors' contributions

MRS cloned viral fragments, performed sequence analysis, analyzed the data and prepared the communication. AM and XC cloned the GPCMV BACs. AM cloned individual genes for sequence analysis. AM, XC and KYC, performed sequence analysis, participated in data analysis, and helped in preparation of the communication. MAM cloned viral DNA fragments, performed sequence analysis, participated in BAC cloning, and aided in preparation of the communication. SVD performed comparative genomic analyses and comparisons and aided in the preparation of the communication.

## Supplementary Material

Additional file 1 ORFs of ≥ 25 aa (tab A). 50 aa (tab B), or 100 aa (tab C) with Blast analysis against other sequenced CMV genomes; e-value cutoff of 0.1. Click here for file

Additional file 2 ORFs of ≥ 25 aa (tab A). 50 aa (tab B), or 100 aa (tab C) with Blast analysis against other sequenced CMV genomes; e-value cutoff of 1e^-5^. Click here for file

Additional file 3 Phylogenetic trees for glycoproteins gB, gH, gO, gL, gM and gN, IRS 1–3 family, and GP116 (functional homolog of UL119; Fc receptor/immunoglobulin binding domains). Alignments generated using both ClustalW and Muscle, as described in the text. Click here for file
