# Supplementary material for: Analysis of the nucleotide sequence of the guinea pig cytomegalovirus (GPCMV) genome
Source: Virol J. 2008 Nov 12;5:139. doi: 10.1186/1743-422X-5-139 (PMC2614972; doi:10.1186/1743-422X-5-139)
Supplement: Additional file 3 — Phylogenetic trees for glycoproteins gB, gH, gO, gL, gM and gN, IRS 1–3 family, and GP116 (functional homolog of UL119; Fc receptor/immunoglobulin binding domains). Alignments generated using both ClustalW and Muscle, as described in the text. [file 1743-422X-5-139-S3.pdf]

gB

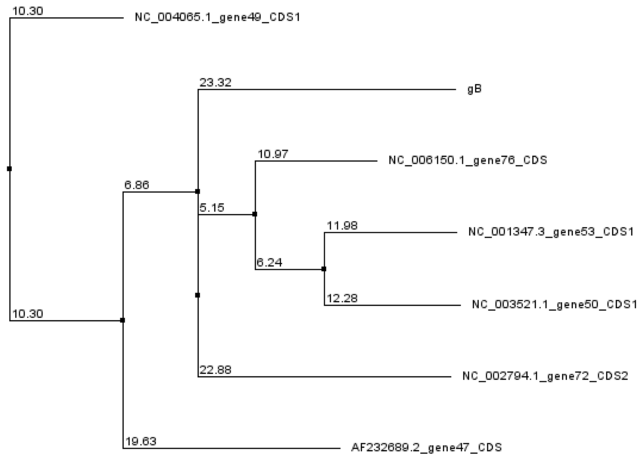

ClustalW

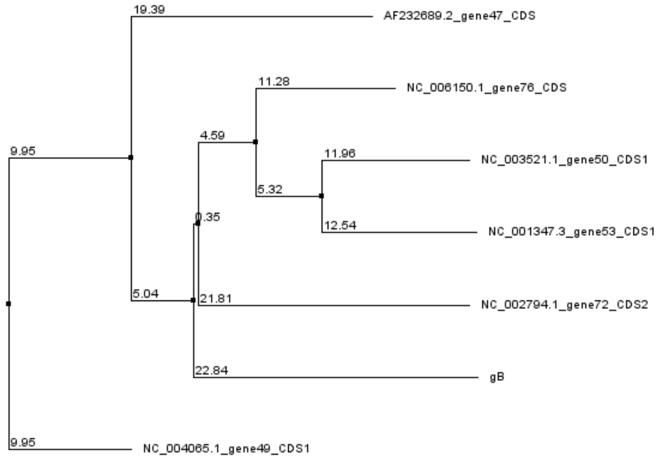

Muscle

gH

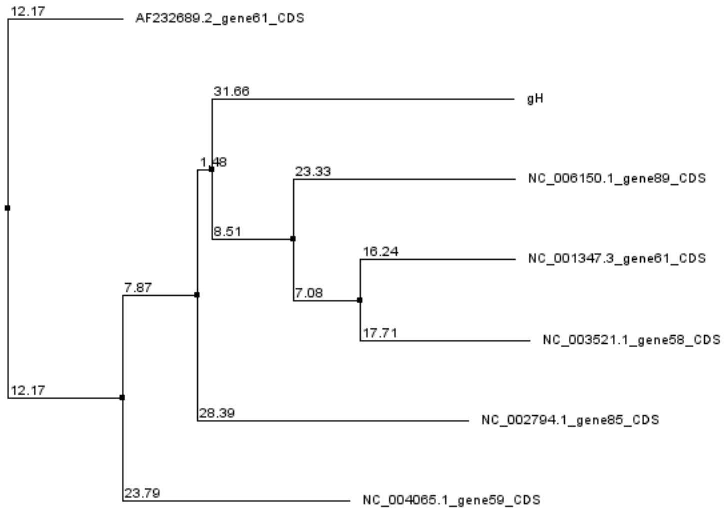

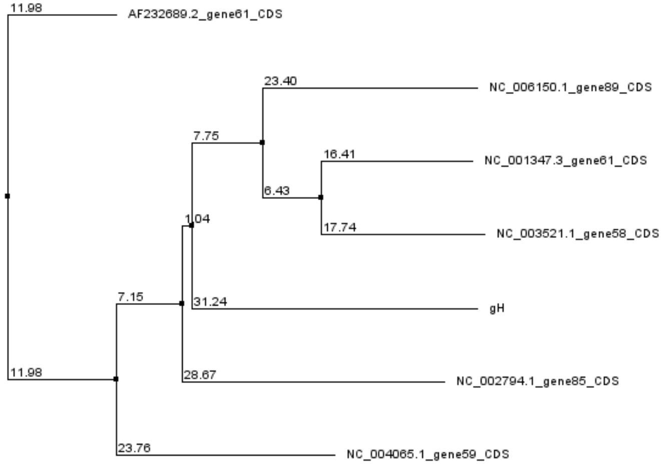

ClustalW

Muscle

gL

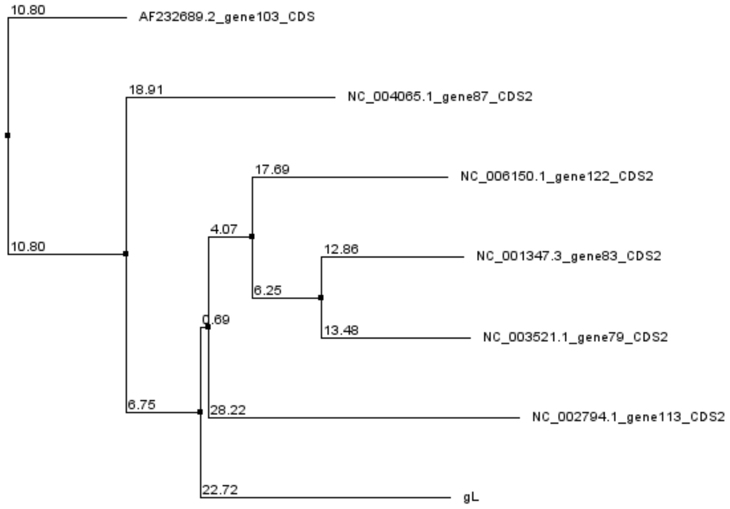

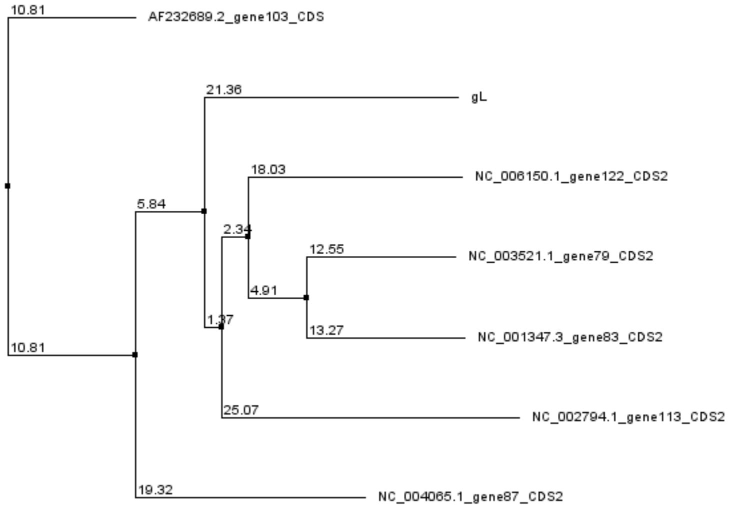

ClustalW

Muscle

gN

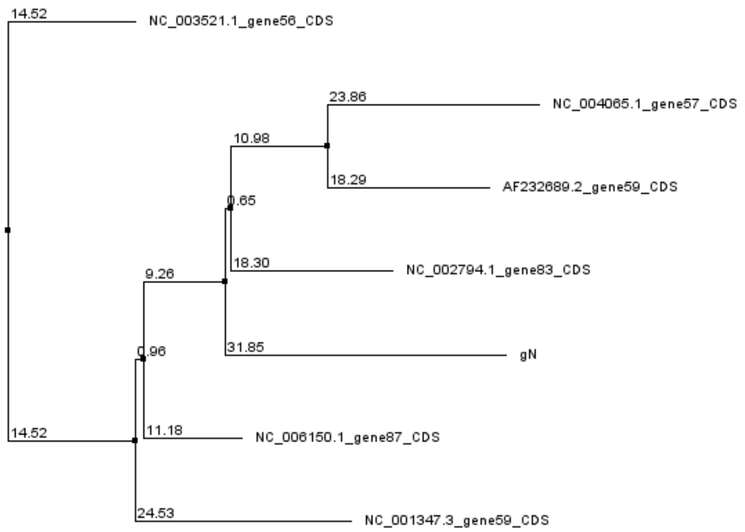

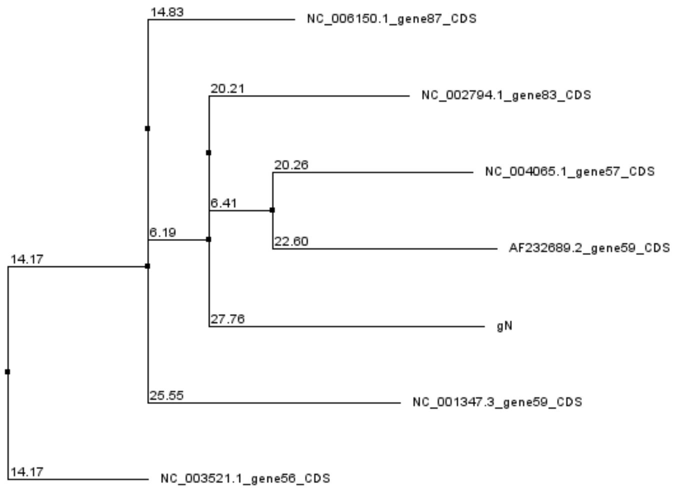

ClustalW

Muscle

gO

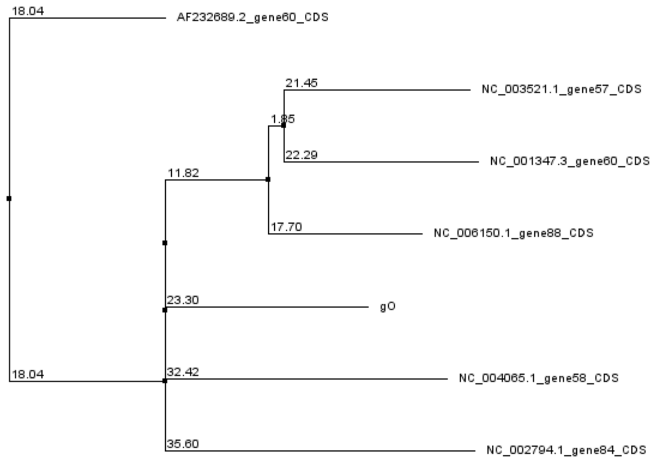

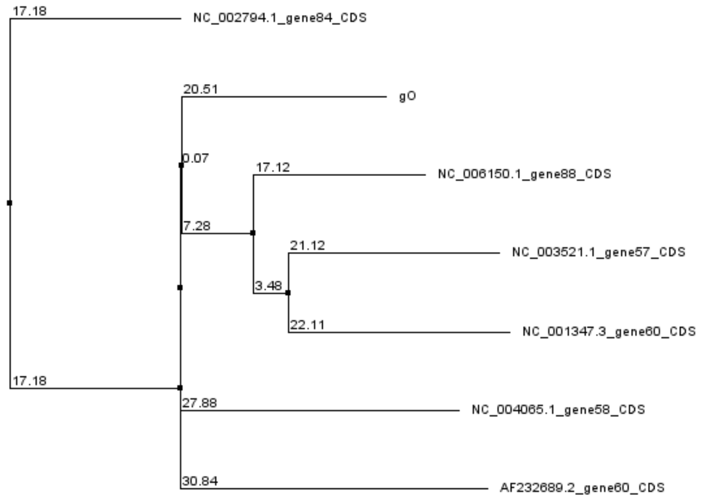

ClustalW

Muscle

gp116

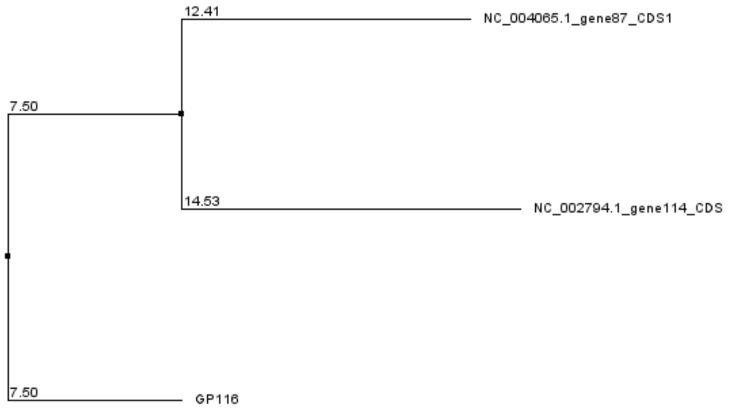

ClustalW

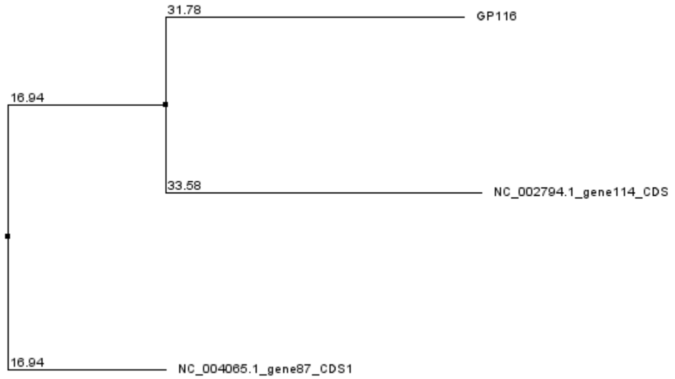

Muscle

irs1

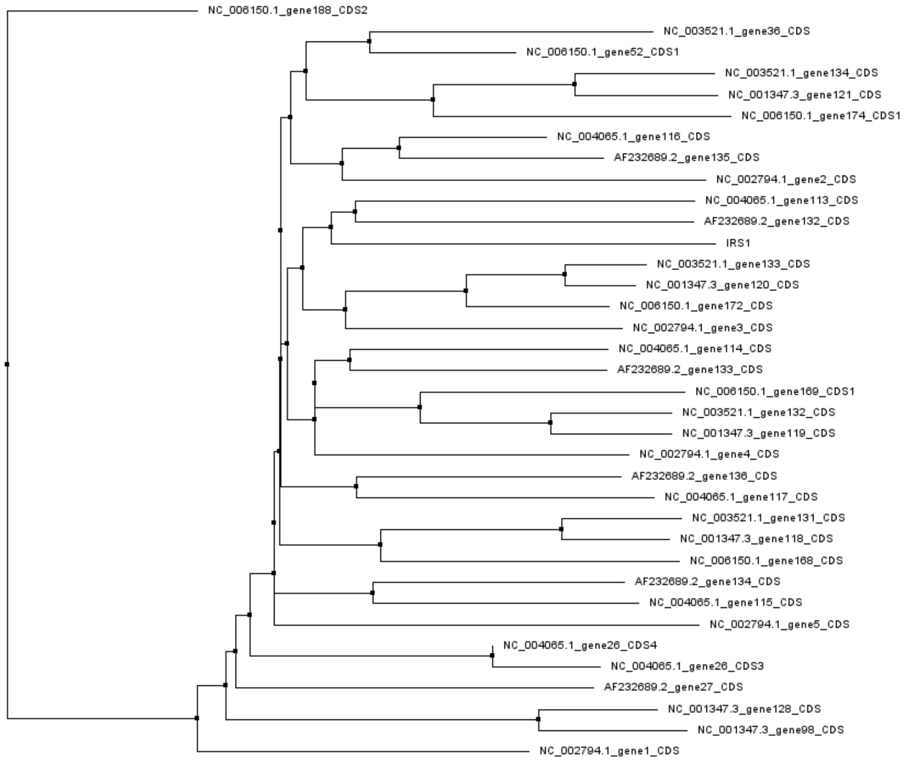

ClustalW

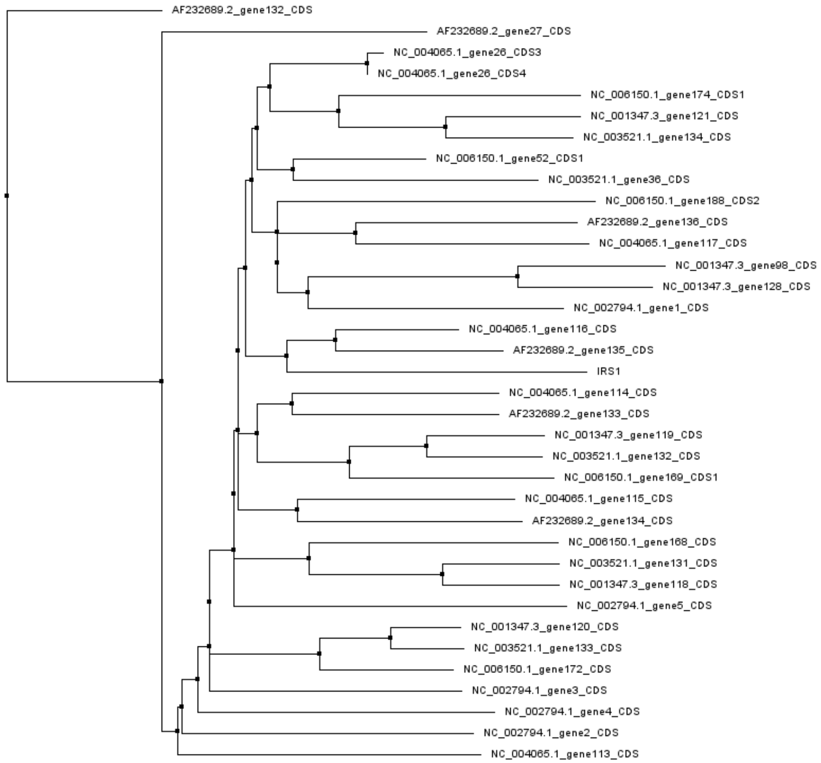

Muscle

irs2

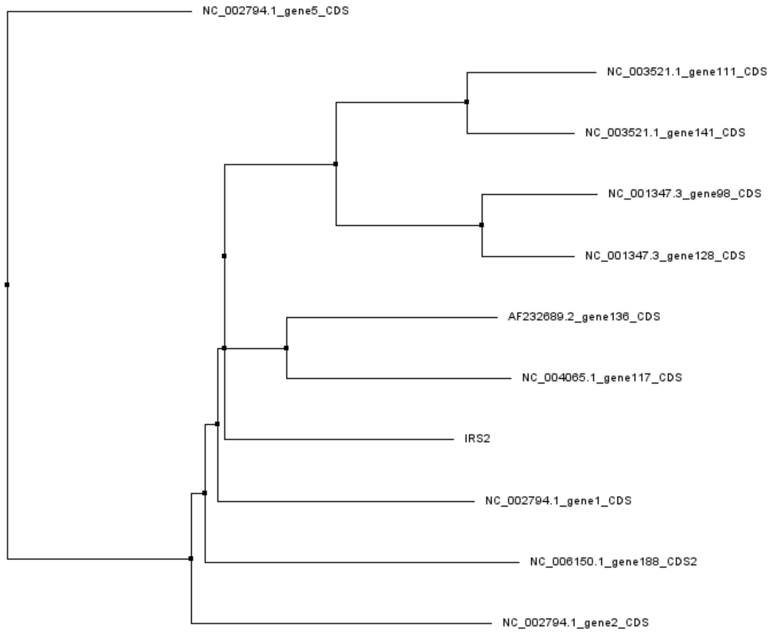

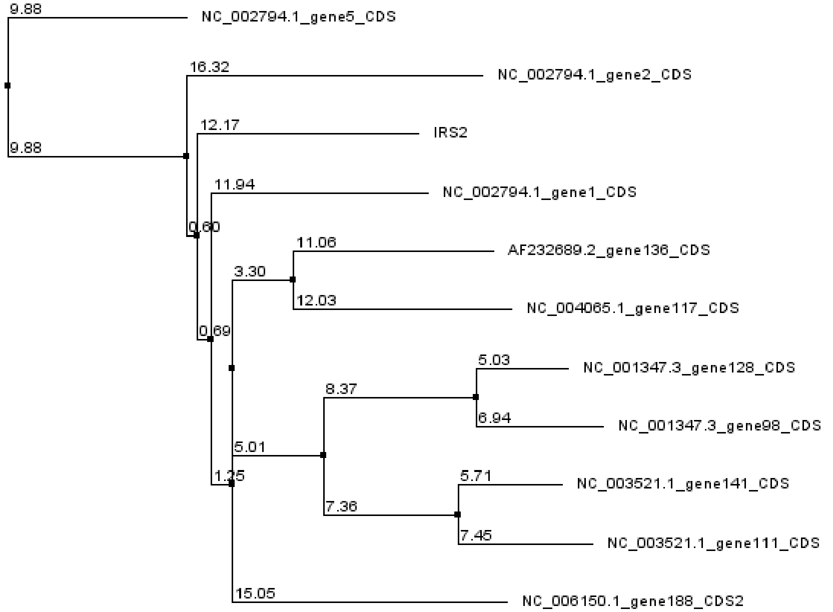

ClustalW

Muscle

irs3

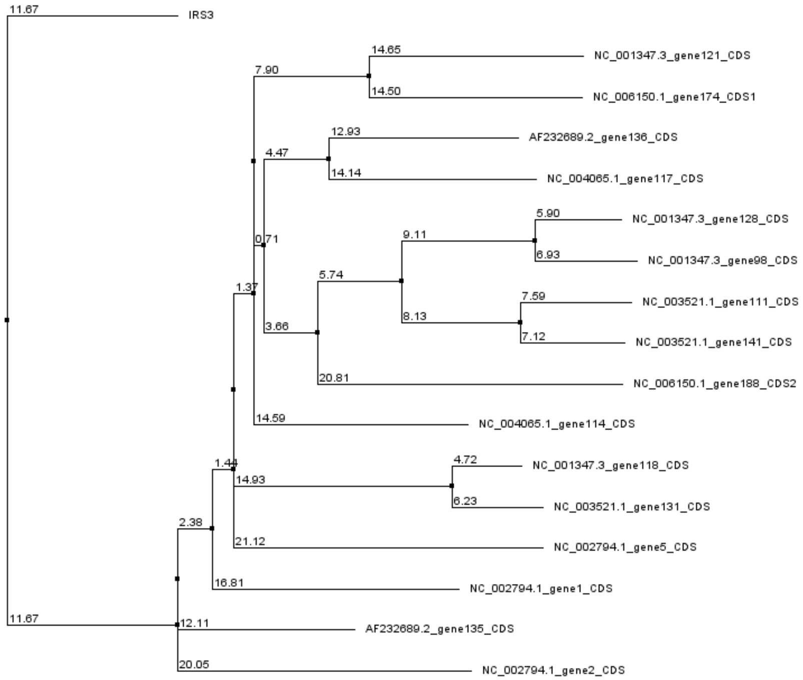

ClustalW

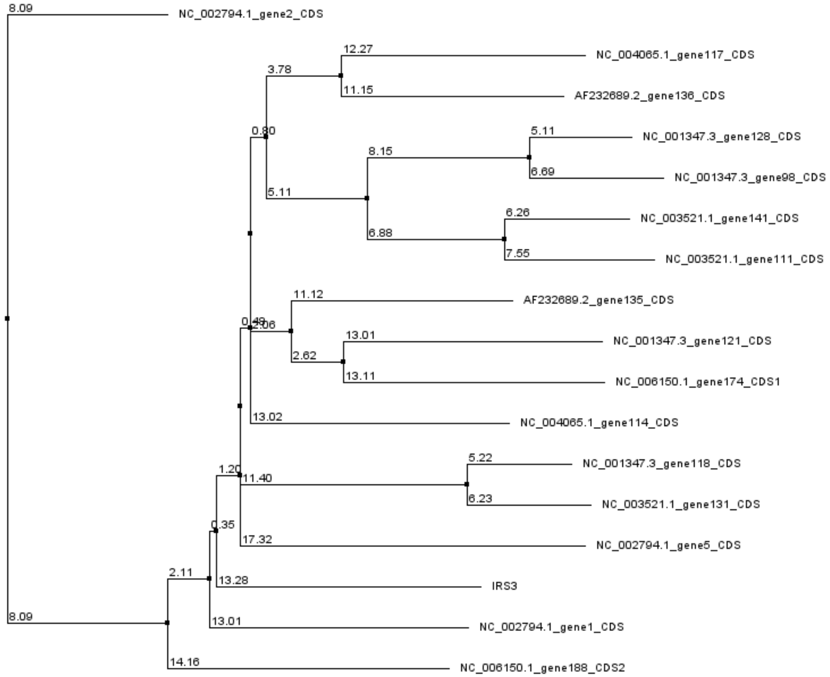

Muscle
